# Ancient association between cation leak channels and Mid1 proteins is conserved in fungi and animals

**DOI:** 10.3389/fnmol.2014.00015

**Published:** 2014-03-07

**Authors:** Alfredo Ghezzi, Benjamin J. Liebeskind, Ammon Thompson, Nigel S. Atkinson, Harold H. Zakon

**Affiliations:** ^1^Department of Neuroscience, University of Texas at AustinAustin, TX, USA; ^2^Department of Integrative Biology, University of Texas at AustinTX, USA; ^3^Marine Biological Laboratory, The Josephine Bay Paul CenterWoods Hole, MA, USA

**Keywords:** *Drosophila*, Cch1, RNAi, Social Space Index, Circadian Rhythms, phenocopy

## Abstract

Neuronal resting potential can tune the excitability of neural networks, affecting downstream behavior. Sodium leak channels (NALCN) play a key role in rhythmic behaviors by helping set, or subtly changing neuronal resting potential. The full complexity of these newly described channels is just beginning to be appreciated, however. NALCN channels can associate with numerous subunits in different tissues and can be activated by several different peptides and second messengers. We recently showed that NALCN channels are closely related to fungal calcium channels, which they functionally resemble. Here, we use this relationship to predict a family of NALCN-associated proteins in animals on the basis of homology with the yeast protein Mid1, the subunit of the yeast calcium channel. These proteins all share a cysteine-rich region that is necessary for Mid1 function in yeast. We validate this predicted association by showing that the Mid1 homolog in *Drosophila*, encoded by the *CG33988* gene, is coordinately expressed with NALCN, and that knockdown of either protein creates identical phenotypes in several behaviors associated with NALCN function. The relationship between Mid1 and leak channels has therefore persisted over a billion years of evolution, despite drastic changes to both proteins and the organisms in which they exist.

## Introduction

Ion channels are the workhorses of neuronal excitability. By controlling the passage of ions across the cell membrane of neurons, ion channels produce and propagate action potentials that transmit information throughout the brain. The best studied families of neuronal ion channels are the ligand-gated and voltage-gated ion channels. These channels initiate, shape, and propagate action potentials (Hille, 2001). In general, these channels are usually closed at the resting membrane potential, but they open and close in response to neurotransmitters or changes in membrane potential. The gating dynamics of these channels strongly affects neural physiology and influences behavioral processes. However, not all neuronal ion channels are closed at the resting potential. One example is the NALCN (Na^+^ leak channel, nonselective) channel. This channel has garnered growing interest because of its involvement in the regulation of neural excitability underlying rhythmic behaviors (Ren, 2011). A gene encoding a sodium leak channel was not demonstrated until 2007 (Gilon and Rorsman, 2009). These channels allow the passive flow of sodium (and other cations) across the membrane and are thought to regulate the activity of rhythmically firing neurons through their modulation of the neuronal resting potential (Lu et al., 2007). They enhance excitability because the Na^+^ that they leak into the cell depolarizes the membrane potential shifting it toward the activation threshold of voltage-gated Na^+^ channels (Na_v_). An increase in the activity of NALCN channels can boost the gain of the neuron, which in turn increases the likelihood that the neuron will fire. These effects can cause a pacemaker neuron to fire continuously.

NALCN channels play an evolutionarily-conserved role in the production of rhythmic neuronal activity and rhythmic behavior. The activity of NALCN channels has been linked to a wide variety of disparate rhythmic behaviors in different species. Examples include rhythmic breathing in mice (Lu et al., 2007), crawling in *C. elegans* (Pierce-Shimomura et al., 2008), and circadian rhythms in flies (Nash et al., 2002). Although these channels are insensitive to voltage, their activity can be modulated by neurotransmitters and calcium via association with accessory proteins (Ren, 2011). Identification and characterization of novel regulators of NALCN channel expression and function are crucial for understanding how nervous systems adapt and maintain appropriate excitability in the face of environmental insults that promulgate abnormal neural activity. However, little is currently known about how NALCN is gated via interaction with its subunits, and it has even been suggested that it is not actually a channel, but rather a sensor that affects membrane permeability indirectly (Senatore et al., 2013). Most of the information about NALCN and its subunits originate from forward genetic screens in model organisms, but comparative genomics offers a powerful alternative for answering these outstanding questions.

Animal NALCN channels are most closely related to fungal calcium channels, and the clade containing both types diverged from a common voltage-insensitive channel ancestor before the diversification of voltage-gated calcium and sodium channels (Liebeskind et al., 2012). Like NALCN, fungal calcium channels have been shown to rely completely on accessory proteins for their function, distinguishing these two channel types from other channels in the voltage-gated super family. The yeast calcium channel, Cch1, relies on a protein called Mid1. Knockout of either protein in yeast creates an identical “mating induced death” phenotype, for which Mid1 was named (Iida et al., 1994). In animals, NALCN channels interact with different proteins in different tissues, including UNC-79, UNC-80, and the M3 muscarinic receptors (M3Rs) (Humphrey et al., 2007; Lu et al., 2009; Swayne et al., 2009; Lu et al., 2010; Lear et al., 2013).

The most parsimonious explanation for the complete reliance on accessory proteins in NALCN and fungal calcium channels is that this was a feature of the channel that was ancestral to both. We reasoned that some elements of this ancient interaction may have been preserved in animals and fungal channels, and that what is known about the interaction between the Cch1 calcium channel in yeast and its accessory protein Mid1, may be used as a model for NALCN function in animals. We show that fungal Mid1 has homologs in animals which most likely interact with NALCN or are involved in the same pathways. This finding highlights the potential of comparative genomics for suggesting functional relationships in difficult molecular systems.

## Materials and methods

### Bioinformatics, conservation profiling, and alignments of Mid1

We initially used BLASTp to search for homologs of Mid1 in animals in the RefSeq database. While BLASTp is sufficient to find a significant hit in insects, the more sensitive search algorithms PSI-BLAST and HMMER were needed to identify the Mid1 domain-containing protein in most other animal lineages. Conservation analyses were performed on the AL2CO server (Pei and Grishin, 2001) and are based on an alignment performed with the E-ins-i algorithm on the MAFFT alignment server (Katoh et al., 2005), as was the alignment in Supplementary figure S1. These alignments were then stripped of majority gapped columns before conservation analyses using GapStreeze (Los Alamos HIV Sequence Database: http://www.hiv.lanl.gov/content/sequence/GAPSTREEZE/gap.html). The programs used for secondary structure prediction are described in Supplementary figure S2 and Supplementary results and discussion.

### Pearson correlation and gene clustering analysis

High-Throughput Gene expression data for the genes *na*, *CG33988*, *unc79*, *unc80* (*CG18437*), *fz4*, and *Act5C*, were obtained from the *Drosophila* database FlyBase (Marygold et al., 2013). These data were collected by the modENCODE project (Celniker et al., 2009) from RNA-Seq analysis of distinct *Drosophila* samples. The modENCODE Tissue Expression Dataset consisted of RNA-Seq analysis of a collection of mRNA samples from 29 different dissected tissues from third-instar larvae and adults. The modENCODE Temporal Expression Dataset used RNA-Seq analysis of a collection of whole animal mRNA samples from 30 distinct developmental stages (Graveley et al., 2011). Pearson's Correlation coefficients of the tissue and developmental gene expression datasets for each gene was performed using GraphPad Prism for Mac software (GraphPad Software, Inc. La Jolla, CA).

### Fly stocks and RNAi gene silencing

*Drosophila* stocks were raised on standard cornmeal agar medium in a 12/12 h light/dark cycle. For all assays, newly eclosed flies were collected over a two-day interval and incubated at 25°C for 5 days before behavioral analysis. During the incubation period, flies were reared in bottles with standard food at all times as mixed genders to allow mating. Flies were separated by gender the day prior to each experiment. The following lines were used for crosses: the pan-neural *elav*-Gal4 line (P{GawB}*elav*^*C*155^ *w*^1118^; P{UAS-*Dcr2*.D}2; +); the UAS-RNAi lines *na*^*JF*01826^ (*y*^1^*v*^1^; +; P{TRiP.JF01826}attP2) and *CG33988*^HMS03014^ (*y*^1^ sc^*^ *v*^1^; +; P{TRiP.HMS03014}attP2/TM3 *Sb*^1^); and the isogenic host strain for the RNAi library *w*^1118^ (*w*^1118^; +; +). All stocks were obtained from the Bloomington Drosophila Stock Center at Indiana University (Bloomington, IN). For RNAi induction, males from each UAS-RNAi line were crossed to the virgin females from the *elav*-Gal4 line and the male progeny (P{GawB}*elav*^*C*155^ *w*^1118^; P{UAS-*Dcr2*.D}2/+; UAS-RNAi/+) tested. Each of the RNAi line and the *elav*-Gal4 line were also crossed to the *w*^1118^ isogenic host strain. The male progeny of these crosses (*w*^1118^; +; UAS-RNAi/+) and (P{GawB}*elav*^*C*155^ *w*^1118^; P{UAS-*Dcr2*.D}2/+; +) were used as controls and tested for comparison. The *elav*-Gal4/UAS-RNAi males that we examined carry the *w*^1118^-bearing X chromosome inherited from their mothers. They did not inherit the *y*^1^ *v*^1^-bearing X chromosome from their fathers. As a result, the *w*^1118^-bearing X chromosome is an important genetic control but the *y*^1^ *v*^1^-bearing X chromosome is not a relevant genetic control. Quantitative RT-PCR analysis of mRNA abundance of the respective knockdown lines indicates that the mode of action of the knockdown mechanism for each gene may be different. Induction of RNAi against *na* results in *na* specific mRNA degradation, but the RNAi against *CG33988* fails to induce *CG33988* mRNA degradation (Supplementary figure S3). This is not surprising, as in some cases RNAi mechanisms involve translational repression of target genes rather than mRNA degradation (Valencia-Sanchez et al., 2006). In fact, the difference in design between the transgenic RNAi constructs strongly supports this possibility (Supplementary results and discussion).

### Diurnal locomotion analysis

Diurnal activity was measured in a DAM2 Drosophila Activity Monitor (Trikinetics Inc., Waltham, MA) using standard procedures (Rosato and Kyriacou, 2006). Briefly, individual flies from each cross were loaded into 5 mm × 65 mm glass tubes. One end of the tube contained a food solution composed of 5% sucrose and 2% agar and sealed with wax. After a fly was loaded into the tube, a piece of cotton was inserted at the other end to prevent the fly from escaping. The tubes were placed into the Drosophila Activity Monitors that records a signal when a fly crosses an infrared beam. The loaded Drosophila Activity Monitors were then placed in a 25°C incubator and maintained in a 12/12 h light/dark cycle. Locomotor activity was recorded every 5 min for 7 days. Data from flies that did not move at least once in any 24-h period were discarded. Analysis of rhythmic locomotive activity was performed using Microsoft Excel and the daily averages plotted in 30-minute bins.

### Social clustering analysis

Social clustering was monitored by introducing 40 male flies of each genotype in horizontal circular chambers as described by Simon et al. (2012) with minor modifications. Chambers consisted of inverted plastic Petri dish lids (90 mm of internal diameter and 7 mm deep) covered with a glass plate. After a 30-minute habituation period, digital images of the chambers were captured from above every 30 min for 2 h (a total of 5 images were capture for every genotype). All genotypes were monitored simultaneously in adjacent chambers. Digital images were imported into ImageJ software version 1.45 s (NIH, rsbweb.nih.gov/ij/) and analyzed for nearest neighbor distances. Flies in each chamber were rendered as particles and assigned 2D coordinates. These coordinates were used to determine the distance from each fly to its nearest neighbor using the Nearest Neighbor macro developed by Yuxiong Mao: https://icme.hpc.msstate.edu/mediawiki/index.php/Nearest_Neighbor_Distances_Calculation_with_ImageJ. The nearest neighbor distances were exported to Prism 5 (GraphPad Software, Inc. La Jolla, CA) for frequency distribution analysis. Distances were divided into 5 mm bins, and the percentage of flies in each bin was calculated for each chamber. The average of the 5 images captured for every chamber was used to determine the frequency distribution of each population. Social space index (SSI) was determined by calculating the difference between the percentage of flies in the first bin (0–5 mm from the nearest neighbor) and the percentage of flies in the second bin (5–10 mm from the nearest neighbor) as described by Simon et al. (2012). A total of 8 replicates were performed for each genotype tested using independent populations. All data are presented as mean ± s.e.m. For comparisons to controls, One-way Repeated Measures ANOVA with Dunnett's *post-hoc* test for multiple comparisons to a control group was used. For all data, differences were considered significant when *P* < 0.05. All social clustering experiments were performed between 3 and 6 pm. In our 12/12 h light/dark protocol, night time (lights-off) starts at 8 pm.

## Results

### Identification of putative NALCN accessory proteins

**Figure 1:**
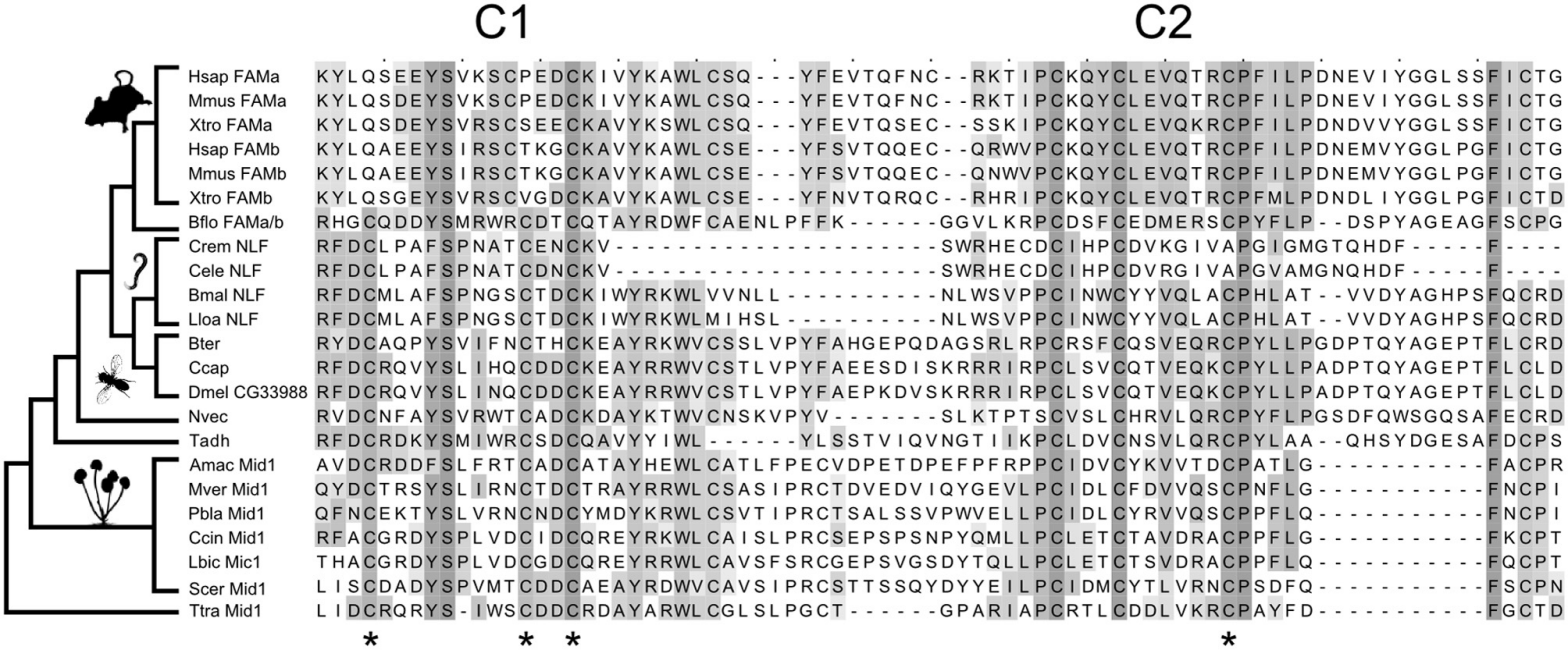
**Evolution of the Mid1 superfamily**. Key cysteine residues are conserved between fungal and animal Mid1. Stars are placed under four cysteine residues that are necessary for Mid1 function in yeast (Maruoka et al., 2002). Three of the four are conserved in fungi, vertebrates and *Caenorhabditis*, and all four are conserved in all other sampled animals. The sampling of the key outgroups to vertebrates (*Branchiostoma floridae*) and *Caenorhabditis* (*Brugia malayi and Loa loa*) allows us to reconstruct the loss of key cysteines and confidently assign homology. The species sampled, from top to bottom are: Vertebrates: Hsap, *Homo sapiens*; Mmus, *Mus musculus*; Xtro, *Xenopus tropicalis*. Chordates: Bflo, *Branchiostoma floridae*. Nematodes: Crem, *Caenorhabditis remanei*; Cele, *Caenorhabditis elegans*; Bmal, *Brugia malayi*; Lloa, *Loa loa*. Insects: Bter, *Bombus terrestris*; Ccap, *Ceratitis capitata*; Dmel, *Drosophila melanogaster*. Basal Animals: Nvec, *Nematostella vectensis*; Tadh, *Trichoplax adhaerens*. Fungi: Amac, *Allomyces macrogynus*; Mver, *Mortierella verticillata*; Pbla, *Phycomyces blakesleeanus*; Ccin, *Coprinopsis cinerea okayama*; Lbic, *Laccaria bicolor*; Scer, *Saccharomyces cerevisiae*. Apusozoa: Ttra, *Thecamonas trahens*. The alignment is shaded by conservation, according to BLOSUM 64 matrix, and has had majority-gapped columns removed. Thumbnails were obtained from PhyloPic.org.

We used protein BLASTp and the more sensitive search algorithms PSI-BLAST and HMMER to search for animal homologs of Mid1 (Altschul et al., 1997; Finn et al., 2011). BLASTp searches of animal genomes identified a group of insect proteins with significant similarities to Mid1. This family includes the *Drosophila* gene *CG33988*. We later noted that these proteins are identified as Mid1 in the public databases RefSeq and PFAM, both of which use HMMER for annotations, and the *Drosophila* gene database FlyBase, which uses InterPro (Hunter et al., 2012). Mid1 had been previously described as having no animal homologs (Iida et al., 1994), and the similarity was weak, but several bioinformatics-based validations support a one-to-one homologous relationship of the insect and fungal proteins with Mid1 domains (Supplementary results and discussion). Further bioinformatics analyses revealed weak, but significant relationships between fungal Mid1, *CG33988*, the NLF-1 genes in nematodes, the FAM155A and B genes in vertebrates, and several uncharacterized proteins in basal animals, suggesting ancient origins of the family (Figure 1 and Supplementary results and discussion).

We found no conserved fungal homologs of either Unc79 or M3R. Searches with Unc80 identified some fungal proteins with local similarity, but these were mostly due to local concentrations of hydrophobic residues in both proteins and were not found in many fungal genomes. Thus, the association of NALCN with these proteins arose later in evolutionary time than the ancient association with Mid1.

### Structure of Mid1

Mid1 is a ~500aa protein that is present in most fungal genomes (Iida et al., 1994). It has been reported as having 4 hydrophobic domains (H1-H4) which are necessary for localization to the membrane (Iida et al., 1994) and 2 cysteine-rich regions (C1 and C2) toward the C-terminus that are hypothesized to act in protein binding (Maruoka et al., 2002). C1 and C2 contain five cysteine residues each and resemble toxins whose complex tertiary structure relies on disulfide bridges between cysteine residues (Possani et al., 1999). Serial deletions suggest that four cysteines in C1 and C2 are essential in yeast (Maruoka et al., 2002), but it is not known how highly conserved Mid1 structure is in other fungi. We therefore made an alignment of 89 Mid1 homologs from across the fungal tree, and plotted conservation scores for each site (Supplementary figure S1). C1, C2, and all 10 cysteine residues are highly conserved; much more so, in fact, than the rest of the gene, suggesting stronger purifying selection at this region of the protein. The C-terminal region may therefore be the main functional zone of Mid1 in fungi.

The animal Mid1-like proteins only align with fungal Mid1 in the highly conserved C1 and C2 regions. Moreover, the most highly conserved regions of C1 and C2 in fungi are precisely those that are conserved in animals (Figure 1). The four essential cysteines are conserved in the insect sequences and three of the four in *Caenorhabditis* NLF-1 and vertebrate FAM155A and B (Figure 1). There is an additional insertion that moves the second essential cysteine N-terminal in vertebrate FAM155. Despite these changes, sampling of cephalochordate and nematode outgroups allows us to assign homology and reconstruct these changes with confidence (Figure 1). We therefore refer to this cysteine-rich area as the Mid1 domain.

### Expression profiles of *Drosophila* Mid1 and NALCN are highly correlated

We hypothesized that the conservation of the cysteine-rich domain between the fungal Mid1 and the *Drosophila* Mid1 protein predicts conservation at the functional level as well, and that Mid1 regulates the activity or expression of the NALCN channel in *Drosophila*, encoded by the gene *na*. To test this possibility, we first examined expression data of the genes that encode the *Drosophila na* and the putative *Drosophila* Mid1 protein encoded by the gene *CG33988*, using modENCODE data from tissue samples and developmental series experiments (Celniker et al., 2009).

**Figure 2:**
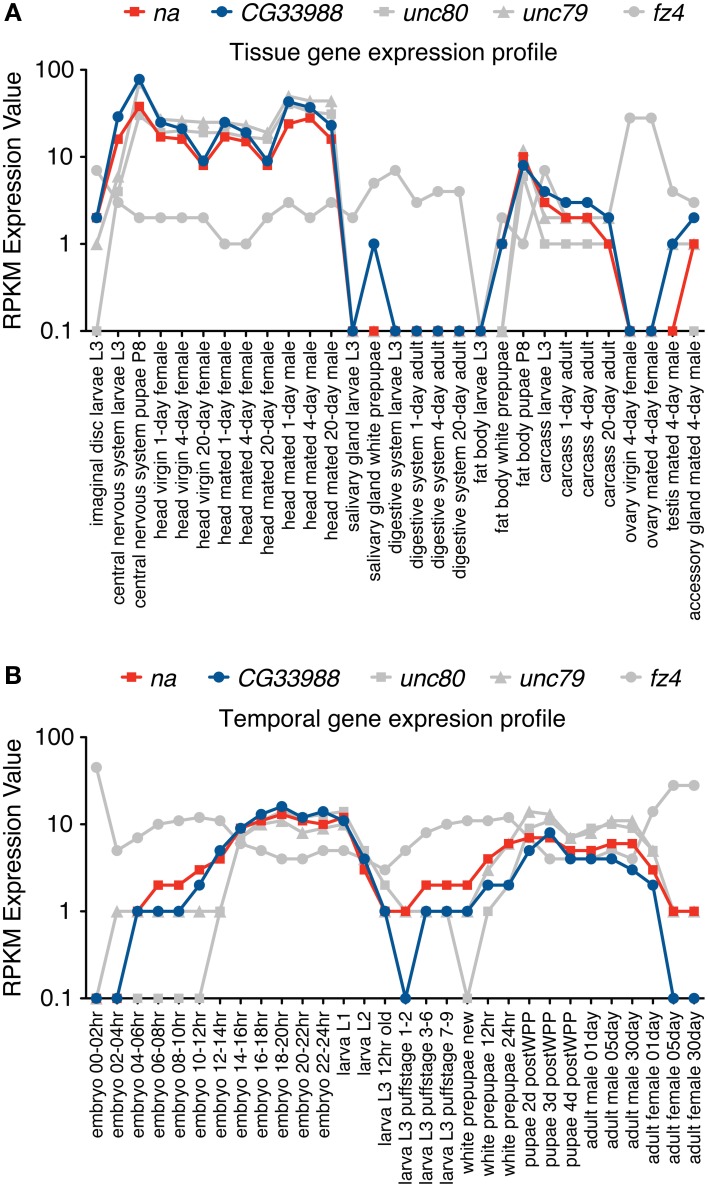
**RNA-Seq tissue and Developmental gene expression profiles for *na* and related genes**. Shown are normalized RPKM (reads per kilobase per million mapped reads) expression values for the genes *na*, *CG33988*, *unc79*, *unc80* and *fz4*, extracted from a collection of 29 different dissected tissues from third-instar larvae and adults **(A)** and 30 distinct developmental stages **(B)** from the modENCODE *Drosophila* Transcriptome project. RNA-Seq coverage data was intersected with FlyBase exons based on the gene model annotations of the current *Drosophila* genome release, to calculate a single value reflecting average coverage per kb per gene. Each gene data point was then classified into one of eight expression level bins, and the graphical summaries were produced from the binned values. RPKM expression values are plotted in a base 10 logarithmic scale.

Gene expression profiles from 5 different genes were examined using these two gene expression datasets. In our analysis we included the gene expression profiles of *na* and *CG33988*, as well as those from 3 other potentially functionally related *Drosophila* genes for comparison. These are the genes *unc79* and *unc80* (*CG18437*), which have been previously linked to *na* and are regarded as potential accessory subunits (Humphrey et al., 2007; Lu et al., 2010; Lear et al., 2013); and the gene *frizzled 4* (*fz4*), which has also been identified as a potential homolog of the yeast Mid1 (Pei and Grishin, 2012). The tissue gene expression data (Figure 2A) shows a clear gene expression correlation between *na* and *CG33988*, *unc79* and *unc80* but not *fz4*. Closer inspection shows that the tissues with the highest transcript levels of the *na* gene accessory members include the central nervous system of larvae and pupae and the head of adult flies, whereas the lowest expression is found in digestive and reproductive tissues. This is consistent with a role in excitable cells.

The developmental series data (Figure 2B) reveal a similar trend. The genes *na*, *CG33988*, *unc79* and *unc80* showed a highly correlated temporal gene expression pattern, strikingly different from that of *fz4*. The *na* group displays two distinct peaks of gene expression; the first spanned from the 8 h embryo to the early larval stage (L1); and the second spanned from the beginning of the pupae stage to adulthood.

To further determine the level of gene expression correlation between *na* and *CG33988*, we performed Pearson correlation analysis using both gene expression profile datasets on all pair wise combinations of these genes, as well as to the other putative *na* accessory genes. This time the expression profiles of the *Act5C* gene were also included as a non-related example. This analysis confirms that in both cases (tissue and developmental expression patterns) the expression patterns of the genes *na*, *CG33988*, *unc79* and *unc80* are highly correlated. In addition, the *fz4* and *Act5C* genes show little or no correlation with *na*, or the other 3 genes (Table 1). Most importantly, for both gene expression profiles, the *na*-*CG33988* are just as highly correlated as the known subunits *unc79* and *unc80*. These data suggest that *na, CG33988, unc79* and *unc80* are co-regulated and participate in the same processes or share common functional roles.

**Table 1:**
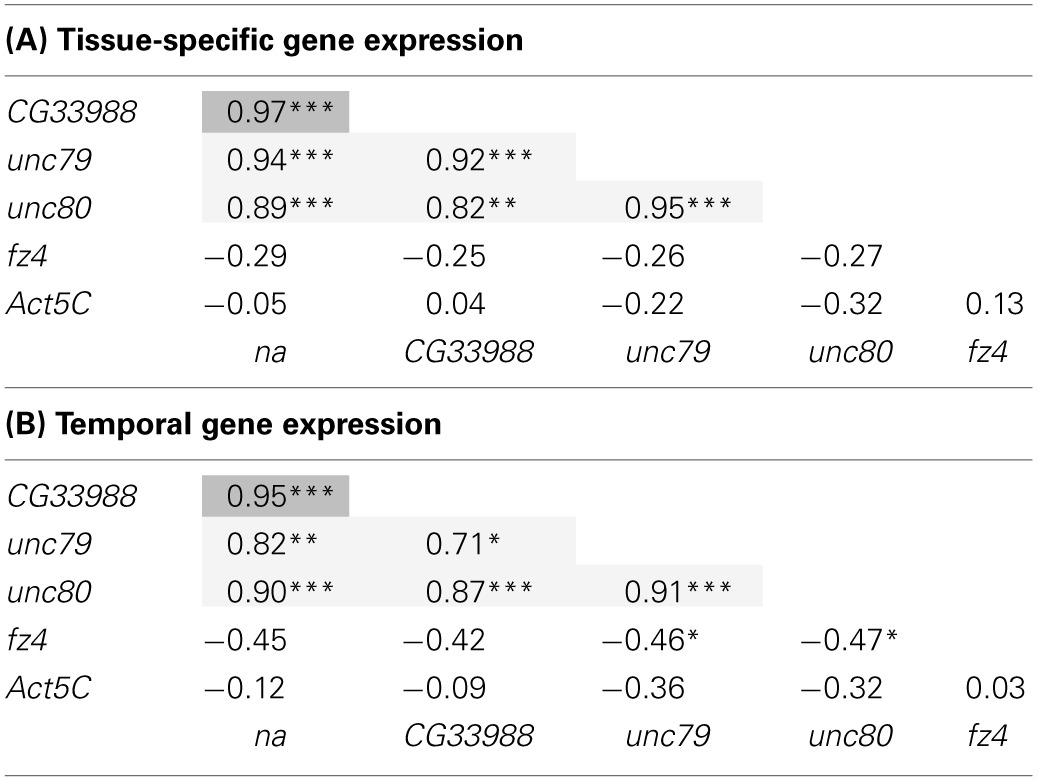
**Gene expression correlation analysis of *na* and related genes**.

Pearson correlation tests performed on D. melanogaster gene expression data from tissue ***(A)*** and developmental series ***(B)*** experiments show that the expression profiles of the na gene and its potentially associated genes are highly correlated. The expression profile of the CG33988 gene exhibits the highest correlation with the na gene (shaded in dark gray). All other highly correlated gene pairs (r > 0.7) are shaded in light gray. Gene names are shown at the left and bottom of the matrix. The gene Act5C is included as a non-related example. Pearson correlation coefficients (r) are displayed. Pearson correlation coefficient p-values: ^***^ < 1e-09, ^**^ < 1e-06, ^*^ < 1e-03, all others values are not significant.

Further gene expression comparisons to other ion channels and synaptic protein genes indicate that the high tissue and developmental correlation coefficients extends to several neuronally expressed genes (Supplemental Dataset S2). These genes include the *Shal* and *Shaker* potassium channel genes, the sodium channel gene *para*, the synaptic active zone component gene encoded by *brp* and the muscarinic acetylcholine receptor gene *mAChR* that is also associated with NALCN in mammals (Swayne et al., 2009). Thus, the expression of *na* and *CG33988* are tightly co-regulated, suggesting interaction, but the expression data may not be fine-grained enough to be considered evidence for a true genetic interaction. We therefore tested whether knockdown of *na* and *CG33988* affected behaviors known to be associated with *na* function.

### RNAi knockdown of *CG33988* phenocopies na knockdown in *Drosophila*

To determine if the *na* and *CG33988* genes have related function, we assayed for genetic interactions using a behavioral genetic approach. If the *Drosophila CG33988* and *na* genes participate in the same physiological or behavioral processes, then one would expect that changes in the expression of either gene will affect similar processes. We investigated the effect of RNAi-induced knockdown on two behaviors known to be affected by *na* in flies: circadian locomotion and social clustering. The *Drosophila* Gal4/UAS expression system was used to drive targeted expression of Gal4 responsive UAS-RNAi constructs against *na* or *CG33988* (Brand and Perrimon, 1993; Dietzl et al., 2007). For all experiments we used a pan-neural Gal4 driver (*elav*-Gal4) that expresses primarily within the neural tissue of the fly (Agrawal et al., 2005).

**Figure 3:**
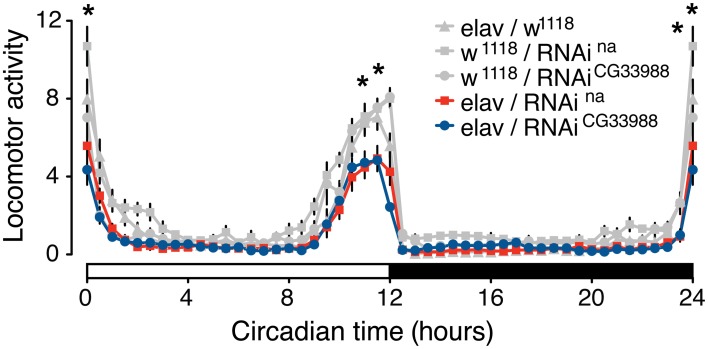
**Targeted RNAi knockdown of *na or CG33988* expression disrupts diurnal control of locomotor behavior**. UAS-responsive RNAi against *na* or *CG33988* were pan-neurally driven using the elav-Gal4 driver. Control flies carry the elav-Gal4 driver in the w^1118^ background used for the UAS-RNAi lines, but lacked the UAS-RNAi transgene. The locomotor activity of each genotype was monitored for a 7-day period and daily averages plotted in 30-minute bins. White bar at the X-axis indicates day period, black bar indicates night period. Asterisks indicate statistically significant difference between the induced-RNAi groups and the control groups for the individual 30-min bins (^*^P < 0.05, repeated measures Two-Way ANOVA).

To test for an effect of the RNAi knockdowns on circadian behavior, we monitored the diurnal activity profiles of flies expressing RNAi against *CG33988*, flies expressing RNAi against *na*, and the appropriate parental controls, over a 7-day period of 12/12 h light/dark cycles. Neural *na* RNAi knockdown effectively induces a robust mutant phenotype characterized by a significant reduction of morning activity as well as reduced anticipation of the “lights-off” transition when compared to controls (Figure 3). This result is in concordance with the reported phenotype of *na* loss-of-function mutations affecting daily rhythms in *Drosophila* (Nash et al., 2002). In these mutants, not only are the activity levels during the “lights-on” period severely disrupted and the anticipation of the “lights-off” transition blocked, but flies also exhibit an inversion of relative locomotor activity in light vs. dark. This latter effect is modest even in complete knockout flies (Nash et al., 2002; Lear et al., 2005), and because RNAi induced silencing of gene expression is technically considered to induce gene knockdown rather than a complete knockout, we did not see this inverted behavioral pattern. While we did not see the “light/dark inverted locomotion activity” phenotype in the RNAi knockdown, we did see a significant reduction of activity during both the “lights-on” and the “lights-off” transition. These important diurnal behaviors have been shown to be controlled by the circadian pacemaker neurons as selective *na* rescue in distinct pacemaker neurons influences rhythmicity and timing of behavior (Lear et al., 2005). Circadian activity enables organisms to anticipate daily events, and this anticipatory behavior is clearly on display when the flies predict the “lights-on” and “lights-off” transitions.

To determine if the *CG33988* gene participates in the control of the same circadian behavior as *na*, we also examined the effects of *CG33988* RNAi knockdown on the diurnal activity assay. RNAi knockdown of *CG33988* using the same pan-neural driver results in an identical circadian aberration (Figure 3). These results corroborate our hypothesis of a genetic interaction between the two genes. Interestingly, expression of *na* or *CG33988* does not cycle in a circadian manner in *Drosophila* (Supplementary results and discussion, and Supplementary figure S4).

Although these experiments establish a role for *CG33988* in diurnal locomotor patterns similar to that of *na*, it is possible that disruption of *CG33988* may have effects on the locomotor activity of flies independent of *na*. Impaired locomotion may lead to the same diurnal activity deficits observed in the *na* knockdown and be indistinguishable from a circadian phenotype if mutant flies simply cannot reach the activity peak of control flies. For instance, RNAi knockdown of the synaptic gene *brp* severely impairs locomotor activity of flies (Wagh et al., 2006), and thereby induces a similar locomotor deficit as the one induced by *na* or *CG33988* knockdown (data not shown). The gene *brp* encodes a pre-synaptic active zone component shown to be a critical player of evoked neurotransmitter release at chemical synapses. Also, mutants in the core circadian clock genes *per* or *Clk* also fail to anticipate the “lights-on” and “lights-off” transitions (Allada et al., 1998), showing that this behavior is sensitive to perturbations in several types of genes. Thus, although it has been shown previously that *CG33988* is upregulated in neurons expressing a molecular clock (Nagoshi et al., 2010), and our experiments confirm that it is necessary for circadian motor output, the circadian behavioral experiments cannot show that *na* and *CG33988* are necessarily coupled. To rule out the possibility that generally impaired locomotor ability is confounding interpretation of our behavioral experiments, we decided to test a second behavior that is associated with mutations in *na*, and is relatively robust to locomotor ability.

### RNAi knockdown of *CG33988* phenocopies *na* social clustering phenotype

Another behavioral phenotype recently associated with a mutation in *Drosophila na* is the disruption of social clustering, the natural tendency of animals of the same species to congregate in close proximity within a group (Burg et al., 2013). In flies, social clustering has been observed and quantified using a measure called social space index (SSI) (Simon et al., 2012). After an initial exploration phase, flies will aggregate in one location with a relatively stable distance between flies. At this stage, the “social space” or distance between individuals shows a consistent distribution between each fly and its closest neighbor, with a majority of flies stationed within 5 mm from their closest neighbor. The SSI is based on the values obtained in the histogram representations of the social space, where SSI equals the percentage of flies in the first bin (0–5 mm) minus the percentage of flies in the second bin (5–10 mm). Mutants in *na* have been shown to have a significantly reduced SSI (Burg et al., 2013).

**Figure 4:**
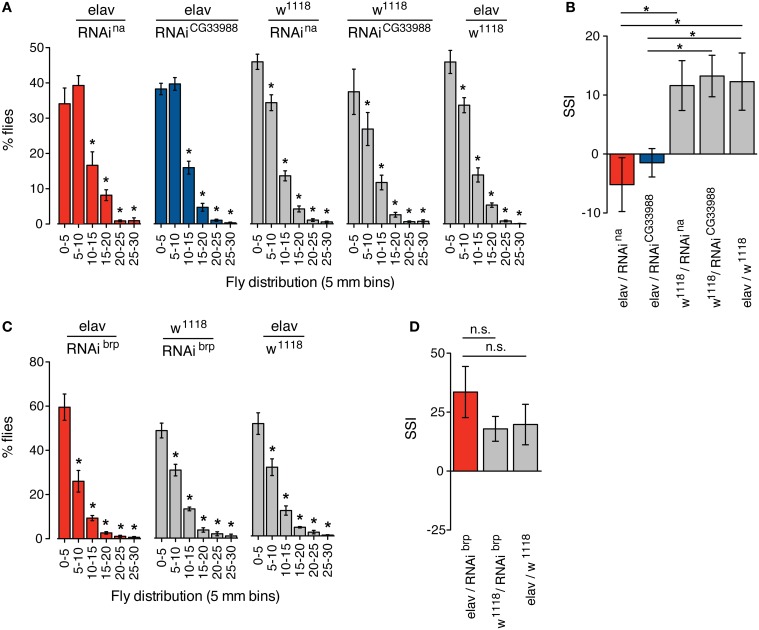
**Targeted RNAi knockdown of *na or CG33988* expression disrupts social clustering behavior**. Quantification of the social space of flies housed in a horizontal circular chamber are shown. **(A)** The histograms display the frequency distribution of the distance between each fly and its closest neighbor expressed as a percentage of the total population, in 5 mm bins, for flies carrying UAS-responsive RNAi against *na* or *CG33988* driven by the pan-neural *elav*-Gal4 driver, or control flies carrying the *elav*-Gal4 driver in the *w*^1118^ background, but lacking the UAS-RNAi transgene, or control flies carrying each UAS-RNAi transgene in the *w*^1118^ background, but lacking the *elav*-Gal4 driver. **(B)** Social Space Index (SSI) calculated for the same groups of flies. **(C)** The histograms display the frequency distribution of the distance between each fly and their closest neighbor expressed as a percentage of the total population, in 5 mm bins, for flies carrying a UAS-responsive RNAi against *brp* driven by the pan-neural *elav*-Gal4 driver and the respective control flies. **(D)** Social Space Index (SSI) calculated for the *brp* RNAi knockdown flies and the respective control flies. Error bars indicate s.e.m. (*n* = 8 trials, 40 males per chamber). Asterisks denote statistically significant differences (*P* < 0.05) based on One-Way Repeated Measures ANOVA with Dunnett's *post-hoc* test for multiple comparisons to the control groups.

We monitored the social space in groups of flies expressing RNAi against *CG33988*, flies expressing RNAi against *na*, and the appropriate transgenic controls. Again, we observed that in both cases neurally induced RNAi knockdown of *na* and *CG33988* similarly and significantly suppressed the social clustering as observed in the frequency distribution of social distances (Figure 4A) and by the reduction in SSI (Figure 4B). We also confirmed that this effect is not a result of a general disruption in locomotor activity, as RNAi knockdown of *brp*, which has severe motor deficits, does not inhibit clustering (Figures 4C,D).

## Discussion

Our results suggest an ancient association between a cysteine-rich motif we call the Mid1 domain and leak channels. This association, which predates the divergence of the animal and fungal lineages, has been preserved in fungal calcium channels and animal NALCN despite structural and functional changes over deep evolutionary time. Our bioinformatics analysis predicted the function of a novel gene in *Drosophila*, *CG33988*, and we validated our prediction using well-characterized behaviors associated with NALCN function. Based on the evident homology between the yeast Mid1 gene and the animal protein family to which *CG33988* belongs, we propose the animal family simply be called *Mid1.*

The cysteine-rich motif that is found in the various Mid1 homologs in animals is the only part of the proteins that is conserved. Furthermore, the secondary structure of the proteins in which the Mid1 domain is found is not always the same (Supplementary results and discussion), and the other parts of the protein do not appear to be homologous. If the secondary structure caused by disulfide bonds between the cysteines is the most critical element of Mid1 function, it may not be surprising that the other areas of the protein are not well conserved.

The loss of key cysteines in vertebrates and *Caenorhabditis*, which are the most divergent homologs we sampled, have apparently not perturbed Mid1's function. In yeast Mid1 localizes to the plasma membrane and endoplasmic reticulum where it is involved in trafficking the calcium channel Cch1 to the plasma membrane (Yoshimura et al., 2004; Hong et al., 2013). A recent study by Xie et al. (2013) showed that *C. elegans* NLF-1 traffics NALCN from the endoplasmic reticulum to the plasma membrane of neurons. Xie et al. found that FAM155A can complement *C. elegans* lines deficient for NLF-1. We have shown that NLF-1, FAM155A, and also its uncharacterized paralog FAM155B, are all members of the Mid1 superfamily. The only regions truly conserved between these genes are the cysteine-rich areas in the Mid1 domain. Thus, an independent functional study in a different model organism confirms our bioinformatic predictions and genetic experiments in *Drosophila*. The data from yeast suggests an ancient function for Mid1 in trafficking Cch1/NALCN.

Our finding that both Mid1 and NALCN are required for a normal circadian motor phenotype in flies fits with earlier studies that have identified *CG33988* in high-throughput screens for circadian associated genes in insects. *CG33988* in flies was found to be up-regulated in neurons that express a molecular clock relative to whole-head and eye (Nagoshi et al., 2010), whereas in bees, the Mid1 homolog was found to cycle in a circadian fashion (Rodriguez-Zas et al., 2012) (Also see Supplementary results and discussion). NALCN is well known to regulate rhythmic behaviors, such as circadian activity patterns, but we find that the association between Mid1 and NALCN extends to social clustering as well. This correspondence, and the evidence that the two genes are coordinately expressed across tissues and developmental stages, suggests a close genetic interaction between NALCN and Mid1, with the former perhaps being trafficked by the latter, as with fungal and nematode Mid1.

The comparative approach offers a powerful method for untangling complicated interactions for which one model system may not meet all requirements. Although this system awaits more concrete evidence for a direct, physical interaction between Mid1 and NALCN, the identification of Mid1 as a superfamily of fungal and animal proteins should enable a fruitful cross-species basis from which to study NALCN function in the future.

### Conflict of interest statement

The authors declare that the research was conducted in the absence of any commercial or financial relationships that could be construed as a potential conflict of interest.
