# Supplementary material for: Ancient association between cation leak channels and Mid1 proteins is conserved in fungi and animals
Source: Front Mol Neurosci. 2014 Mar 7;7:15. doi: 10.3389/fnmol.2014.00015 (PMC3945613; doi:10.3389/fnmol.2014.00015)
Supplement: Supplementary file 2 [file DataSheet2.PDF]

Ancient association between cation leak channels and Mid1 proteins is conserved in fungi and animals

Supplementary material

1. Supplementary materials and methods

1.1. Gene expression analysis by qPCR

Total RNA was extracted from heads of groups of approximately 100 flies (mix of males and females) using TRIzol® RNA Isolation Reagent (Ambion, Austin, TX) and purified using Direct-zol™ RNA MiniPrep Kit (Zymo Research, Irvine, CA). Residual genomic DNA was digested by TURBO™ DNase treatment (Ambion, Austin, TX). First-strand cDNA was synthesized from total RNA using the SuperScript® III reverse transcriptase (Invitrogen/Life technologies, Carlsbad, CA) using a mix of random hexamers (28 uM) and oligo-dT$_{20}$ (50 uM) primers. The cDNAs were amplified by real-time PCR using the TaqMan Universal Master Mix No UNG (Applied Biosystems, Branchburg, NJ) in a Viia7 Real-Time PCR System (Applied Biosystems) following the manufacturer's protocols. Double quenched (FAM, ZEN, 3IABkFQ) hydrolysis probes (IDT, Coralville, IA) were used to analyze relative expression from each gene. Primers and probes were designed to span exon splice junctions using IDT's online software (Table S1). Gene specificity of primers was confirmed by PCR (Takara Bio inc. Japan) followed by direct sequencing of purified bands cut from agarose gel. RT reactions with no reverse transcriptase added were used as a negative control for all RNA samples analyzed. Reverse transcription was performed in triplicate and each cDNA sample had triplicate PCR reactions.

Table S1: Primers and probe used in qPCR analysis.

Gene	Primer / probe sequence 5'→ 3'	
na	Probe:	**56-FAM**-CGTGCGCGA-ZEN-TGTTCTGATCAAGG-**3IABkFQ**
	Sense:	GGTGCTCTCATTCAAGGGATG
	Antisense:	ACGAACAAAGTCAGACCGATC
CG33988	Probe:	**56-FAM**-TAGGCAGAG-ZEN-AAATGTCGGCTCGC-**3IABkFQ**
	Sense:	AAACCGTGGAACAAAAGTGC
	Antisense:	CCAGTCTCGGGTATGTTTTGA
per	Probe:	**56-FAM**-CGCCTCGAA-ZEN-GACGTTGCACTGCT-**3IABkFQ**
	Sense:	CGGACACCATCGCGTCTT
	Antisense:	GCGCTTCACGATATCCTCCTT
RpL32	Probe:	**56-FAM**-CCTCCAGCT-ZEN-CGCGCACGTTG-**3IABkFQ**
	Sense:	CTGCCCACCGGATTCAAG
	Antisense:	CGATCTCGCCGCAGTAAAC
Cyp1	Probe:	**56-FAM**-CGGCAAGTC-ZEN-CATCTACGGCAACAAGTT-**3IABkFQ**
	Sense:	ACCAACCACAACGGCACTG
	Antisense:	TGCTTCAGCTCGAAGTTCTCATC

Relative expression was determined using LinRegPCR software (Ramakers *et al.*, 2003). A common threshold fluorescence in the exponential phase was used to measure the Cq value for all genes in each genotype for comparison. Exponential phase was determined by fitting a regression to the log-linear phase of each amplification. If the $R^2 > 0.99$ and there were at least 4 points then the slope of the regression was used to estimate doubling efficiency for that reaction. The doubling efficiency was taken as the average of all the reactions (Karlen *et al.*, 2007): 3 technical replicates and 3 RT replicates in the RNAi study and 4 technical replicates in the circadian rhythm study. If the average efficiency was within 5% of 2 then the ΔΔCt method was used to calculate relative expression (Livak and Schmittgen, 2001). The expression of each gene was normalized by the geometric mean of two stable housekeeping genes: *RpL32* and *Cyp1* (Vandesompele *et al.*, 2002). Statistical significance was calculated using the One-way ANOVA for each gene with Dunnett's post-hoc test for comparisons to the induced RNAi sample.

2. Supplementary results and discussion

2.1. The cysteine-rich domain is unique to Mid1-like proteins.

The primary sequence similarity between fungal and animal Mid1 was weak enough to warrant in-depth bioinformatics validations, which we describe here. All BLAST and PSI-BLAST searches were initially validated using a reciprocal best BLAST approach. This criterion is true for fungal Mid1 and all animal orthologs except those in *Caenorhabditis* (NLF-1), which lack a critical cysteine in the C2 region of the gene. The obvious orthology between *Caenorhabditis* NLF-1 and *Brugia* and *Loa* NLF-1 fills in the necessary information to place NLF-1 within the Mid1 superfamily, however. Two paralogous Mid1 orthologs were found in vertebrates: FAM155A and B (Figure 1). These likely duplicated after the evolution of vertebrates, because the lancelet *Branchiostoma floridae* has only a single copy (XP_002593356.1). They also have an insertion that shifts the second critical cysteine over by one amino acid. This insertion is not present in *Branchiostoma* (Figure 1), suggesting that it occurred specifically in vertebrates, but before the gene duplication. Xie et al. (Xie *et al.*, 2013) found that FAM155A could complement the function of NLF-1 in *C. elegans*, but did not identify FAM155B or other Mid1 orthologs in animals. We predict that FAM155B, and perhaps any other animal ortholog, may be able to complement NLF-1 function.

While the Blastp e-values were typically above 0.01 (RefSeq database) due to the small region of alignment, we noted that the max-identity was typically around 60% between insects and yeast. We investigated the possibility that the similarity of the animal genes to Mid1 in BLAST analyses may be an artifact due to poor alignment in other regions, and that the cysteine-rich motif may be common in other, unrelated genes. We used three different methods: 1) PSI-BLAST searches of animal genomes using yeast Mid1 as a query and a position-specific score matrix (PSSM) generated by a PSI-BLAST search of fungal genomes with Mid1. The PSSM would then represent the most conserved regions amongst Mid1 homologs in fungi and would guide the search based on this profile. 2) BLAST searches of animal genomes with just the C-terminal region of yeast Mid1, and 3) HMMER searches using a hidden Markov model built from a large alignment of fungal Mid1 homologs that had been stripped down to just the C-terminal region. These searches were performed on the NCBI's server in the non-redundant protein database, or on the HMMER server, with the same database.

The same insect proteins were identified as among the top matches in all searches. The C1 and C2 regions did not have clear similarity to other documented protein families. Repeated iterations of PSI-BLAST and HMMER searches did find some similarity to the frizzled proteins, as previously reported (Pei and Grishin, 2012), but this is likely due to convergence, rather than homology.

These methods also identified proteins in the early-branching animals *Trichoplax adhaerens* (XP_002116197.1), and the sea anemone *Nematostella vectensis* (XP_001626959) that resembled the C1 and C2 of Mid1. Another match was found in the oomycete genus *Phytophthora* (*P. infestans* - XP_002904056.1; *P. sojae* - EGZ07722.1). Oomycetes are plant pathogens that resemble fungi but are distantly related, and the presence of these genes in *Phytophthora* suggests horizontal gene transfer, which has been documented between these groups (Richards *et al.*, 2006). *Phytophthora* also contain four-domain ion channels that group with the voltage-insensitive clade (Liebeskind *et al.*, 2012), but this channel is completely uncharacterized and whether it might be regulated by this Mid1-like protein is a matter of speculation. A Mid1 domain has also been found in the apusozoan protist *Thecamonas trahens* ((Cai and Clapham, 2012), Figure 1). This group is thought to be sister to opisthokonts, the group that includes fungi and animals.

The presence of proteins with Mid1 domains in early-branching metazoans like *Trichoplax* and still more in apusozoa suggests deep homology between animal proteins and fungal Mid1, with loss and primary sequence divergence across animal and fungal phyla.

2.2. Structure prediction

We then looked for similarities in secondary structure between the fungal and insect Mid1 proteins. Contrary to earlier findings, we did not find four transmembrane segments in *S. cerevisiae* Mid1 (Supplementary figure S2). MEMSAT SVM, a support vector-based algorithm, predicts a transmembrane segment just C-terminal of C2 in *D. melanogaster* and coincident with C2 in *S. cerevisiae*, suggesting similar structure in the two proteins. Phobius, a HMM-based prediction algorithm used by the PFAM server predicts a transmembrane segment in *D. melanogaster* consistent with the MEMSAT prediction, but does not predict one in *S. cerevisiae*. Another HMM-based algorithm, TMHMM, predicted no transmembrane segments in either protein. Xie et al. (Xie *et al.*, 2013) found a structure for *C. elegans* NLF-1 that is consistent with the predictions reported in Supplementary figure S2, but the predictions for the other proteins should be considered provisional.

2.3. qPCR analysis of RNAi gene silencing

Throughout this study, we have used targeted RNAi knockdown to specifically block activity from the *na* and *CG33988* genes. In order to assess the mode of action and efficacy of the knockdown mechanism, we measured the mRNA abundance of the *na* and *CG33988* genes in flies undergoing targeted RNAi knockdown and in control animals.

Quantitative RT-PCR analysis of the *na* mRNA abundance showed that pan-neural induction of RNAi against *na* results in a significant and highly specific decrease in *na* mRNA levels (Supplementary figure S3A). Elav-Gal4 induction of an RNAi transgene targeting the *na* gene (elav/RNAi^{na}) results in at least a 3-fold reduction in expression in comparison to all of the control

groups. Furthermore, the *na* mRNA levels of flies carrying the elav-Gal4 induced RNAi transgene targeting the *CG33988* gene (elav/RNAi^{CG33988}) remained at control levels. On the other hand, analysis of the mRNA abundance *CG33988* showed more ambiguous results (Supplementary figure S3B). Elav-Gal4 induction of the RNAi transgene targeting the *CG33988* gene (elav/RNAi^{CG33988}) shows no significant changes in the CG33988 mRNA levels, when compared to most controls. A modest (~25%) but significant reduction in expression was only seen in comparison to the elav-Gal4-only control (elav/w^{1118}). These results clearly indicate that the RNAi against *na* results in *na* mRNA degradation, but the RNAi against *CG33988* fails to induce *CG33988* mRNA degradation. We believe however, that these results do not invalidate the effectiveness of the RNAi knockdown for silencing genes, as in some cases RNAi mechanisms may actually involve the translational repression of target genes rather than mRNA degradation (Valencia-Sanchez *et al.*, 2006).

It is important to note that the transgenic RNAi lines used to knockdown *na* and *CG33988* use slightly different RNA interference mechanisms. While both lines were obtained from the Transgenic RNAi Project (TRiP) at Harvard University, they were generated using different design approaches. The UAS-RNAi^{na} was generated using the first generation VALIUM10 - TRiP vector, carrying a long double-stranded RNA hairpin fragment. The hairpin was designed as a 468bp long fragment of the *na* transcript sequences that is in common to all splice-forms of the gene. Processing of the long double-stranded RNA hairpin results in short interfering RNAs (siRNAs) that lead to mRNA degradation. Meanwhile, the UAS-RNAi^{CG33988} was generated using the second generation VALIUM20 - TRiP vector, designed to deliver a single 21bp siRNA against *CG33988* using the endogenous microRNA (miRNA) pathway. The difference in design between these two transgenic RNAi methods, may in fact lead to distinct silencing mechanisms. While the canonical RNAi pathway elicited by long double-stranded RNA hairpin fragment result in mRNA degradation of target genes, the endogenous microRNA pathway often acts through translational repression with little or no influence on mRNA abundance (Bethune *et al.*, 2012). This difference can certainly account for the contrasting results obtained.

Fly lines generated by crossing tubulin-Gal4 flies with the UAS-RNAi^{CG33988} lines were found to be lethal. Tubulin is expressed highly across tissues, and the fact that knocking down *CG33988* on this wide scale is lethal not only suggests a central function for this novel gene, but also that the RNAi induction mechanisms is very effective in knocking down the gene.

2.4. Fly circadian motor output

Circadian behavioral rhythms in Drosophila are determined by a molecular clock that coordinates neural output to the motor system (Allada and Chung, 2010). The molecular clock is expressed in a core set of pacemaker neurons that is in turn broken into subsets that mediate specific behavioral outputs. Lateral ventral neurons (LNvs) mediate the morning anticipation behavioral peak, and lateral dorsal neurons (LNds) and one group of dorsal neurons (DN1) underlie the evening anticipation peak. The resting membrane potential and intrinsic firing rate in large LNvs varies in a circadian fashion, with periods of hyper-excitability corresponding to behavioral activity peaks, suggesting that the molecular clock is transduced into electrical excitability within the core set of neurons (Sheeba *et al.*, 2010).

Although the molecular underpinnings of the circadian change in resting membrane potential are not known, the NALCN channels have been shown to be critical for neural output of the Drosophila

circadian pacemaker (Lear *et al.*, 2005). The Drosophila NALCN gene *na* is predominantly expressed in the core set of clock-expressing neurons. While mutations in the gene result in poor circadian rhythms, selective *na* rescue in distinct pacemaker neurons restores rhythmicity and timing of behavior indicating that the gene is necessary for proper circadian function. However, the gene encoding NALCN in flies does not seem to cycle in a circadian fashion (Nash *et al.*, 2002).

The Drosophila *CG33988* gene and its homolog in the honey bee Apis mellifera, have both been implicated in circadian-related processes by high-throughput transcriptomics. In flies, *CG33988* was found to be up-regulated in LNs relative to whole-head and eye (Nagoshi *et al.*, 2010; Kula-Eversole *et al.*, 2010), whereas in bees, the Mid1 homolog was found to cycle in a circadian fashion (Rodriguez-Zas *et al.*, 2012). Here we found that knockdown of Drosophila *CG33988* phenocopies knockdown of the *na* (NALCN) in a circadian behavior experiment, suggesting a close association between the two proteins in this neural system. The fact that *na* does not cycle in flies, but *CG33988* cycles in bees and is expressed in LNs in flies suggests that *CG33988* may be the protein that couples the molecular clock to the changes in excitability in motor circuits. However, when we tested this hypothesis using qPCR analysis of circadian mRNA expression, as with *na,* we did not detect any circadian cycling of the Drosophila *CG33988* gene (Supplementary figure S4). This result suggests that circadian control of resting membrane potential occurs via other or less direct means.

3. Supplementary references

Allada, R., and Chung, B. Y. (2010). Circadian organization of behavior and physiology in Drosophila. *Annu. Rev. Physiol.* 72, 605-624. DOI: 10.1146/annurev-physiol-021909-135815

Bethune, J., Artus-Revel, C. G., and Filipowicz, W. (2012). Kinetic analysis reveals successive steps leading to miRNA-mediated silencing in mammalian cells. *EMBO Rep.* 13, 716-723. DOI: 10.1038/embor.2012.82

Cai, X., and Clapham, D. E. (2012). Ancestral Ca2+ signaling machinery in early animal and fungal evolution. *Mol. Biol. Evol.* 29, 91-100. DOI: 10.1093/molbev/msr149

Kall, L., Krogh, A., and Sonnhammer, E. L. (2004). A combined transmembrane topology and signal peptide prediction method. *J. Mol. Biol.* 338, 1027-1036. DOI: 10.1016/j.jmb.2004.03.016

Karlen, Y., McNair, A., Perseguers, S., Mazza, C., and Mermod, N. (2007). Statistical significance of quantitative PCR. *BMC Bioinformatics* 8, 131. DOI: 10.1186/1471-2105-8-131

Kula-Eversole, E., Nagoshi, E., Shang, Y., Rodriguez, J., Allada, R., and Rosbash, M. (2010). Surprising gene expression patterns within and between PDF-containing circadian neurons in Drosophila. *Proc. Natl. Acad. Sci. U S A* 107, 13497-13502. DOI: 10.1073/pnas.1002081107

Lear, B. C., Lin, J. M., Keath, J. R., McGill, J. J., Raman, I. M., and Allada, R. (2005). The ion channel narrow abdomen is critical for neural output of the Drosophila circadian pacemaker. *Neuron* 48, 965-976. DOI: 10.1016/j.neuron.2005.10.030

Liebeskind, B. J., Hillis, D. M., and Zakon, H. H. (2012). Phylogeny unites animal sodium leak channels with fungal calcium channels in an ancient, voltage-insensitive clade. *Mol. Biol. Evol.* 29, 3613-3616. DOI: 10.1093/molbev/mss182

Livak, K. J., and Schmittgen, T. D. (2001). Analysis of relative gene expression data using real-time quantitative PCR and the 2(-Delta Delta C(T)) Method. *Methods* 25, 402-408. DOI: 10.1006/meth.2001.1262

Maruoka, T., Nagasoe, Y., Inoue, S., Mori, Y., Goto, J., Ikeda, M., and Iida, H. (2002). Essential hydrophilic carboxyl-terminal regions including cysteine residues of the yeast stretch-

activated calcium-permeable channel Mid1. *J. Biol. Chem.* 277, 11645-11652. DOI: 10.1074/jbc.M111603200

Nagoshi, E., Sugino, K., Kula, E., Okazaki, E., Tachibana, T., Nelson, S., and Rosbash, M. (2010). Dissecting differential gene expression within the circadian neuronal circuit of Drosophila. *Nat. Neurosci.* 13, 60-68. DOI: 10.1038/nn.2451

Nash, H. A., Scott, R. L., Lear, B. C., and Allada, R. (2002). An unusual cation channel mediates photic control of locomotion in Drosophila. *Curr. Biol.* 12, 2152-2158.

Nugent, T., and Jones, D. T. (2009). Transmembrane protein topology prediction using support vector machines. *BMC Bioinformatics* 10, 159. DOI: 10.1186/1471-2105-10-159

Pei, J., and Grishin, N. V. (2012). Cysteine-rich domains related to Frizzled receptors and Hedgehog-interacting proteins. *Protein Sci.* 21, 1172-1184. DOI: 10.1002/pro.2105

Ramakers, C., Ruijter, J. M., Deprez, R. H., and Moorman, A. F. (2003). Assumption-free analysis of quantitative real-time polymerase chain reaction (PCR) data. *Neurosci. Lett.* 339, 62-66.

Richards, T. A., Dacks, J. B., Jenkinson, J. M., Thornton, C. R., and Talbot, N. J. (2006). Evolution of filamentous plant pathogens: gene exchange across eukaryotic kingdoms. *Curr. Biol.* 16, 1857-1864. DOI: 10.1016/j.cub.2006.07.052

Rodriguez-Zas, S. L., Southey, B. R., Shemesh, Y., Rubin, E. B., Cohen, M., Robinson, G. E., and Bloch, G. (2012). Microarray analysis of natural socially regulated plasticity in circadian rhythms of honey bees. *J. Biol. Rhythms* 27, 12-24. DOI: 10.1177/0748730411431404

Sheeba, V., Fogle, K. J., and Holmes, T. C. (2010). Persistence of morning anticipation behavior and high amplitude morning startle response following functional loss of small ventral lateral neurons in Drosophila. *PLoS ONE* 5, e11628. DOI: 10.1371/journal.pone.0011628

Tada, T., Ohmori, M., and Iida, H. (2003). Molecular dissection of the hydrophobic segments H3 and H4 of the yeast Ca2+ channel component Mid1. *J. Biol. Chem.* 278, 9647-9654. DOI: 10.1074/jbc.M206993200

Valencia-Sanchez, M. A., Liu, J., Hannon, G. J., and Parker, R. (2006). Control of translation and mRNA degradation by miRNAs and siRNAs. *Genes Dev.* 20, 515-524. DOI: 10.1101/gad.1399806

Vandesompele, J., De Preter, K., Pattyn, F., Poppe, B., Van Roy, N., De Paepe, A., and Speleman, F. (2002). Accurate normalization of real-time quantitative RT-PCR data by geometric averaging of multiple internal control genes. *Genome Biol.* 3, RESEARCH0034.

Xie, L., Gao, S., Alcaire, S. M., Aoyagi, K., Wang, Y., Griffin, J. K., Stagljar, I., Nagamatsu, S., and Zhen, M. (2013). NLF-1 delivers a sodium leak channel to regulate neuronal excitability and modulate rhythmic locomotion. *Neuron* 77, 1069-1082. DOI: 10.1016/j.neuron.2013.01.018

4. Supplementary Figures

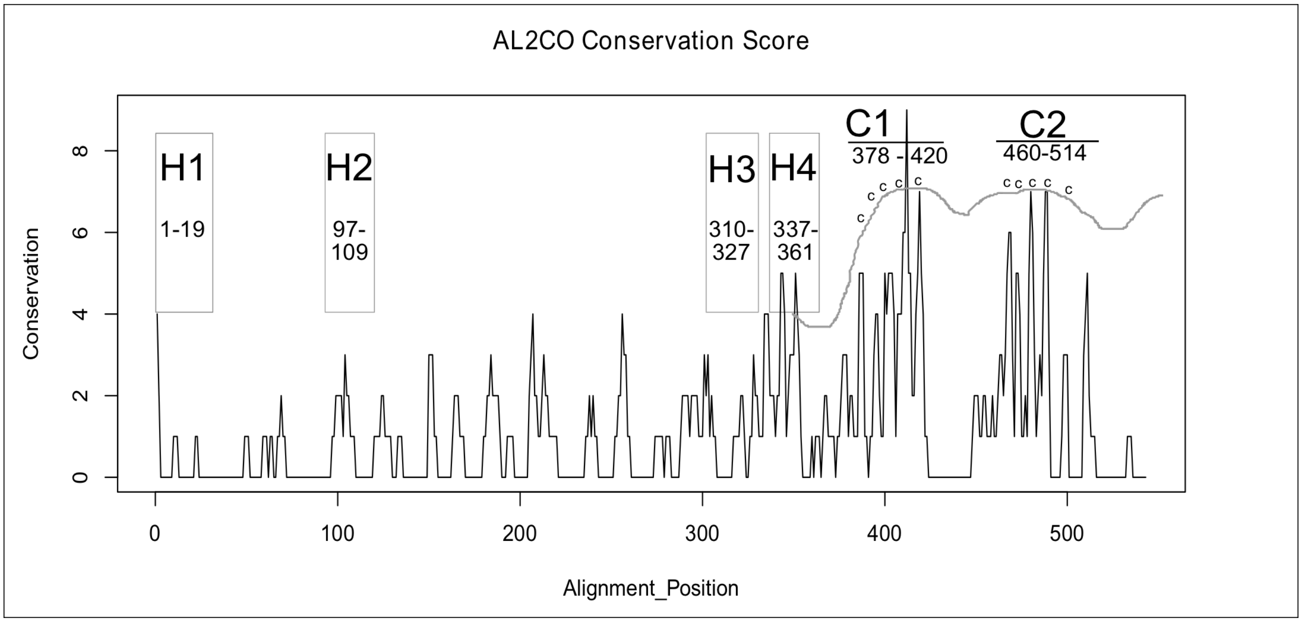

Supplementary figure S1. Conservation profile of 86 fungal Mid1 proteins. Transmembrane segments and the two cysteine-rich regions C1 and C2 as reported in Tada et al. (Tada *et al.*, 2003) are plotted for reference. The analysis was carried out on the AL2CO server. Majority-gapped columns were removed from the alignment before the analysis.

		Transmembrane Prediction Algorithm		
		MEMSAT-SVM	Phobius	TMHMM
S. cerevisiae	C1: 417-450<br>C2: 487-507	482-497	None	None
D. melanogaster	C1: 649-678<br>C2: 881-917	1074-1089	1075-1092	None

Supplementary figure S2: Comparison of structure prediction algorithms. Three different structure prediction algorithms (Nugent and Jones, 2009; Kall *et al.*, 2004) predict different transmembrane structures. None conformed to traditional predictions for Mid1 (Maruoka *et al.*, 2002). One, MEMSAT-SVM, which uses a sophisticated machine learning algorithm to predict structure, predicts a transmembrane segment in analogous places for yeast and fly Mid1, after C2.

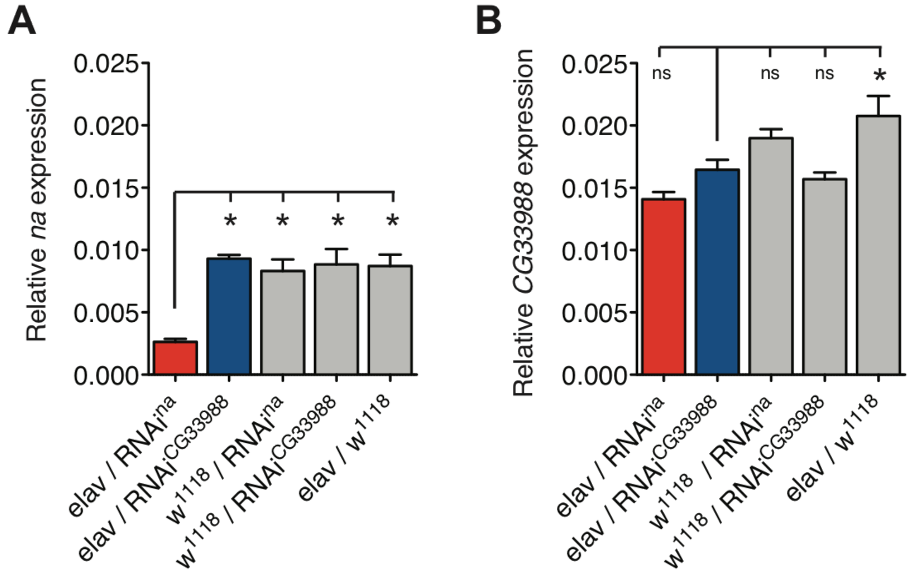

Supplementary figure S3: Quantitative PCR measurement of *Drosophila na* and *CG33988* RNAi knockdown. Relative expression of the *na* and *CG33988* genes as determined by quantitative reverse-transcription PCR analysis. Abundance of mRNA for each gene is expressed relative to the geometric mean of the abundance of the *Cyp1 and RpL32* genes. **(A)** Relative *na* expression in flies carrying inducible RNAi transgenes against *na, CG33988,* or the appropriate controls. Red bar indicate flies with neuronally-induced RNAi against *na* (elav / $RNAi^{na}$). Statistical comparisons between the RNA levels of the elav / $RNAi^{na}$ and the controls was determined by One-way ANOVA with Dunnett's post-hoc test. Asterisks denote P<0.05. **(B)** Relative *CG33988* expression in flies carrying inducible RNAi transgenes against *na, CG33988,* or the appropriate controls. Blue bar indicate flies with neuronally-induced RNAi against *CG33988* (elav / $RNAi^{CG33988}$). Statistical comparisons between the RNA levels of the elav / $RNAi^{CG33988}$ and the controls was determined by One-way ANOVA with Dunnett's post-hoc test. Asterisks denote P<0.05, n=3 for each group.

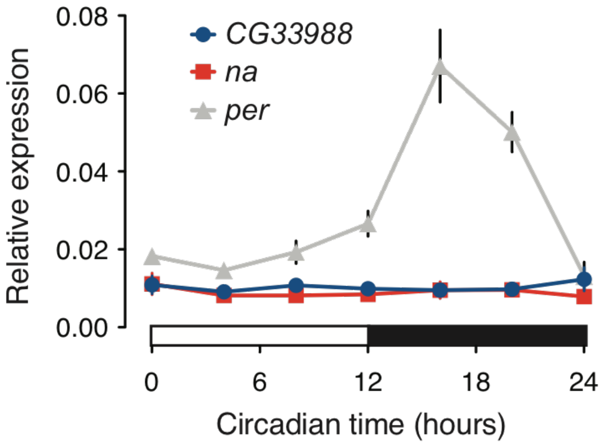

Supplementary figure S4: Circadian expression profile of *Drosophila na* RNAi knockdown. Relative circadian expression of *na*, *CG33988* and the core circadian gene *per* as determined by quantitative reverse-transcription PCR analysis. Abundance of mRNA for each gene is expressed relative to the geometric mean of the abundance of the *Cyp1 and RpL32* genes. Error bars are Standard Deviation, n=4 for each time point.

Dataset S1: Gene expression correlation analysis of *na* and other neuronally expressed genes. Pearson correlation analysis of the gene expression profiles of 16 neurally expressed genes is shown. Gene expression data was collected from tissue and developmental series experiments. The tissue expression profile of the *na* gene exhibits very high correlation with the *CG33988* gene as well as with other ion channel and synaptic genes. The developmental expression profile of the *na* gene also exhibits very high correlation with the *CG33988* gene as well as with other ion channels and synaptic genes. In this case, *na* and *CG33988* share the highest correlation amongst all the genes tested. Pearson correlation coefficients (r) and p-values for each pair of genes are displayed.
